# Rethinking Radiation Therapy for Perineural Invasion in Oral Cancer: Does More Coverage Improve Outcomes?

**DOI:** 10.1002/cam4.71134

**Published:** 2025-08-12

**Authors:** Sarbani Ghosh Laskar, Anuj Kumar, Ashwini Adhau, Shwetabh Sinha, Samarpita Mohanty, Munita Bal, Neha Mittal, Swapnil Rane, Asawari Patil, Ashwini Budrukkar, Monali Swain, Pallavi Rane, Gouri Pantvaidya, Sudhir Nair, Deepa Nair, Anuja Deshmukh, Shivakumar Thiagarajan, Richa Vaish, Vidisha Tuljapurkar, Chandrashekhar Dravid, Poonam Joshi, Rathan Shetty, Arjun Singh, Pankaj Chaturvedi

**Affiliations:** ^1^ Department of Radiation Oncology Tata Memorial Centre, Homi Bhabha National Institute Mumbai India; ^2^ Department of Pathology Tata Memorial Centre, Homi Bhabha National Institute Mumbai India; ^3^ Department of Biostatistics Tata Memorial Centre, Homi Bhabha National Institute Mumbai India; ^4^ Department of Head and Neck Surgery Tata Memorial Centre, Homi Bhabha National Institute Mumbai India

**Keywords:** oral squamous cell carcinoma, perineural invasion, radiation therapy, recurrence patterns, survival outcomes, target volumes

## Abstract

**Introduction:**

Perineural invasion (PNI) in oral squamous cell carcinoma (OSCC) is linked to aggressive tumour behaviour and poorer survival outcomes. Adjuvant radiotherapy (RT) is recommended for PNI‐positive OSCC, but optimal RT target volume remains uncertain.

**Methods:**

This study retrospectively analysed 103 patients with histopathologically confirmed PNI‐positive OSCC treated between January 2017 and December 2023. All patients underwent surgery followed by adjuvant RT, with or without concurrent chemotherapy. Recurrence patterns were categorised as in‐field, marginal or out‐of‐field. Survival outcomes, including overall survival (OS) and disease‐free survival (DFS), were assessed using the Kaplan–Meier method, and prognostic factors were analysed using univariate and multivariate models.

**Results:**

The median follow‐up was 22.2 months. The 2‐year OS and DFS were 63% (95% CI: 53%–75%) and 57% (95% CI: 48%–68%), respectively. In‐field recurrences constituted 70% of local failures, with no recurrences observed at the skull base despite conservative RT volumes. Extensive PNI, large nerve involvement and extratumoral spread were significantly associated with higher recurrence rates and poorer survival. Multivariate analysis identified advanced tumour stage (T3/T4) and extranodal extension (ENE) as independent predictors of worse OS (HR: 2.67, *p* = 0.016; HR: 2.08, *p* = 0.045, respectively), while depth of invasion (DoI) > 10 mm significantly impacted DFS (HR: 0.28, *p* = 0.04 for DoI ≤ 10 mm).

**Conclusion:**

Our findings suggest that expanding RT volumes to cover entire cranial nerve pathways may not improve outcomes and increase the risk of toxicity. A personalised approach to RT planning, incorporating PNI extent, nerve involvement and other high‐risk features, is essential for optimising treatment outcomes in PNI‐positive OSCC.

## 1 Introduction

Oral squamous cell carcinoma (OSCC) is a significant public health concern, particularly in South Asia, where risk factors such as tobacco use, betel quid chewing and alcohol consumption are prevalent [1, 2]. In India, the widespread use of smokeless tobacco products like gutkha and khaini, along with areca nut chewing, has led to a disproportionately high incidence of OSCC. According to GLOBOCAN 2022, over 389,485 new cases of oral cavity cancer were reported worldwide, with India contributing a substantial proportion due to its unique socio‐cultural practices [3]. Despite advances in treatment, survival outcomes remain suboptimal, especially in patients presenting with high‐risk pathological features underscoring the need for optimised therapeutic strategies.

The treatment of OSCC involves a multidisciplinary approach combining surgery, radiotherapy (RT) and chemotherapy. Surgery is often curative for early‐stage disease; however, adverse histopathological features such as DoI, poorly differentiated histology, perineural invasion (PNI), lymphovascular emboli (LVE), worst pattern of invasion (WPOI), close or positive margins and nodal involvement with or without extranodal extension (ENE) necessitate adjuvant therapy to mitigate the risk of recurrence [4]. Among these features, PNI has gained recognition for its prognostic significance, often indicating an aggressive disease phenotype with deeper invasion, higher rates of nodal metastasis and worse survival outcomes [5, 6].

PNI, defined as the invasion of tumour cells into the perineural space [7, 8, 9] includes two histologic patterns as specified by Liebig et al. The first pattern (type A) involves tumour cells located within the peripheral nerve sheath, with infiltration into all three nerve sheath layers. The second pattern (type B) is characterised by tumour cells in close proximity to the nerve, involving at least 33% of its circumference. PNI is further categorised based on focality (focal vs. extensive), nerve size involvement (small vs. large nerves) and location (intratumoral vs. extratumoral), each of which impacts prognosis and treatment decisions [10, 11]. Current guidelines recommend adjuvant RT for PNI‐positive OSCC, often advocating comprehensive cranial nerve coverage. However, the optimal delineation of RT target volumes remains debated, with some guidelines supporting extensive cranial nerve coverage to distal branches, while others favour more conservative approaches to minimise toxicity [12, 13, 14].

Despite these recommendations, the role of adjuvant RT in PNI‐positive OSCC, particularly when PNI is the sole adverse feature, remains unclear. Retrospective studies have yielded mixed results, with some demonstrating improved survival and locoregional control, while others highlight the potential for increased toxicity without clear benefit, reflecting the biological heterogeneity of PNI [15, 16]. This ambiguity underscores the need for a more nuanced understanding of PNI and its implications for RT planning.

Our study aims to address these gaps by evaluating the impact of RT volumes on patterns of failure in PNI‐positive OSCC patients treated at our centre. By analysing recurrence patterns, survival outcomes and the role of PNI subcategories, we seek to provide evidence‐based recommendations for RT target volume delineation. This approach focuses on optimising treatment efficacy while minimising toxicity, thereby enhancing the quality of life for long‐term survivors.

## 2 Methodology

This retrospective observational study was conducted at a tertiary cancer centre in India focusing on patients with OSCC treated between January 2017 and December 2023. Eligible patients included those with biopsy‐proven, non‐metastatic OSCC who underwent surgery and had histopathologically confirmed PNI. All patients underwent surgery followed by adjuvant RT, with or without concurrent chemotherapy, using intensity‐modulated radiation therapy (IMRT) techniques. To maintain uniformity in data collection and analysis, patients treated outside the institution or with incomplete treatment records were excluded.

Data were extracted from institutional electronic medical records and treatment planning systems. Demographic variables included age, gender and lifestyle factors such as tobacco and alcohol use. Clinical details covered tumour subsite, staging per the AJCC 8th edition and PNI characteristics, such as focal versus extensive involvement, intratumoral versus extratumoral spread and nerve size involvement. Surgical records provided information on the extent of resection and reconstructive techniques, while radiotherapy details included target volumes, doses and fractionation schedules. Concurrent chemotherapy details, including indications like ENE or positive surgical margins, were also documented.

Radiotherapy planning scans were reviewed to assess clinical and planning target volumes (CTV and PTV). Where available, preoperative imaging was fused with RT planning scans for target volume delineation. For patients with recurrence, diagnostic imaging at recurrence was fused with the initial RT planning scans with the appropriate registration taken into account to classify the recurrence as in‐field (≥ 95% of the recurrence volume within the 95% isodose line), marginal (20%–95% within the 95% isodose) or out‐of‐field (< 20% within the 95% isodose), as defined by Dawson et al. [17].

### 2.1 Ethical Considerations

The study was approved by the Institutional Ethics Committee (IEC) at Tata Memorial Hospital, Mumbai, India, under approval number OIEC/4316/2024/00002. Given the retrospective nature of this study, considering the minimal risk to participants and the use of de‐identified data, a short consent was taken to update outcomes data when necessary. The study was conducted in accordance with the principles of the Declaration of Helsinki.

### 2.2 Statistical Analysis

Data analysis was performed using SPSS version 25.0 and R Studio (R version 4.0.3). Survival outcomes, including OS and DFS, were calculated using the Kaplan–Meier method. The log‐rank test was employed to compare survival curves between subgroups based on clinicopathological features, such as T‐stage, N‐stage, ENE and PNI characteristics.

Univariate analysis was conducted to identify factors significantly associated with survival outcomes, such as tumour stage, DoI and patterns of PNI. Significant variables from univariate analysis were entered into a multivariate Cox proportional hazards model to assess their independent prognostic value. Hazard ratios (HRs) and 95% confidence intervals (CIs) were reported for each factor. A two‐tailed *p* value of < 0.05 was considered statistically significant.

## 3 Results

A total of 103 patients with OSCC and histopathologically confirmed PNI were included in the analysis. The median age of the cohort was 48 years (range 32–70 years), with 68.9% of patients being under 55 years. Male patients constituted 79.6% of the cohort. Tumour subsites included the tongue and floor of mouth in 57.3% of cases, and bucco‐alveolar complex in 42.7%. Most patients (64.1%) presented with pathological stage IV disease, with T4 tumours comprising 45.6% of the cohort. pN3 disease was observed in 38.8%, indicating extensive nodal metastasis.

DoI exceeded 10 mm in 73.8% of cases, with 18.4% having a DoI between 5 and 10 mm. The WPOI was predominantly categorised as WPOI4 (59.2%), followed by WPOI5 (32%). Lymphovascular invasion was observed in 7.8% of the cohort. ENE was present in 48.5% of patients, with 26.2% exhibiting minor ENE and 14.6% showing major ENE. Surgical margins were negative in 88.3% of patients, while close or positive margins were documented in 11.6%. Table 1 summarises the key demographic, clinical and histopathological characteristics of the study cohort, including adverse risk features.

**TABLE 1:** Demographic and HPR characteristics of the entire cohort.

Category	Group	Number	Percent (%)
Age	< 55 years	71	68.9
	≥ 55 years	32	31.1
Gender	Male	82	79.6
	Female	21	20.4
Habits (tobacco/alcohol)	Yes	80	77.7
	No	23	22.3
Sites	Tongue & floor of mouth	59	57.3
	Buccoalveolar complex	44	42.7
Clinical stage	Stage I	5	4.9
	Stage II	13	12.6
	Stage III	19	18.4
	Stage IV	66	64.1
Pathological stage	Stage I	4	3.9
	Stage II	6	5.8
	Stage III	19	18.4
	Stage IV	74	71.8
Histology	MDSCC	66	64.1
	PDSCC	34	33
	WDSCC	2	1.9
	Sarcomatoid	1	1
LVI	Present	8	7.8
	Absent	94	91.3
	Not Known	1	1
DoI	≤ 5 mm	2	1.9
	> 5–≤ 10 mm	19	18.4
	> 10 mm	76	73.8
WPOI	Not known	6	5.8
	3	3	2.9
	4	61	59.2
	5	33	32
	Not known	6	5.8
PPOI	1	2	1.9
	2	1	1
	3	62	60.2
	4	32	31.1
	5	1	1
	Not known	5	4.9
ENE	Present	50	48.5
	Absent	52	50.5
	Not known	1	1
ENE category	≤ 2 mm (minor)	27	26.2
	> 2 mm (major)	15	14.6
	Not known	61	59.2
Margins	Negative	91	88.3
	Close	6	5.8
	Positive	6	5.8

Abbreviations: DoI, depth of invasion; ENE, extranodal extension; HPR, histopathology report; LVI, lymphovascular invasion; WPOI, worst pattern of invasion; PPOI, predominant pattern of invasion.

### 3.1 Quantification of PNI

The study analysed PNI characteristics based on nerve size, focality and tumour location. Large nerve involvement was observed in 11.7% of cases, while small nerve involvement accounted for 22.3%. Regarding focality, focal PNI was present in 44.7% and extensive PNI in 20.4% of patients. In terms of tumour location, intratumoral PNI was the most common, seen in 52.4%, followed by extratumoral PNI in 6.8%, and a combination of both in 9.7% of cases. Quantification was not available for some patients due to incomplete histopathological data. Table 2 provides a detailed summary of PNI quantification across these parameters.

**TABLE 2:** Quantification of PNI.

PNI type	Number	Percent (%)
Large nerve	12	11.7
Small nerve	23	22.3
Unknown (nerve)	68	66
Focal	46	44.7
Extensive	21	20.4
Unknown (focality)	36	35
Intratumoral	54	52.4
Extratumoral	7	6.8
Both	10	9.7
Not known	32	31.1

Abbreviation: PNI, perineural invasion.

### 3.2 Treatment Characteristics

All patients underwent surgical resection with curative intent, followed by adjuvant radiotherapy (RT) in 88.3% of cases to a standard dose of 60 Gy delivered using IMRT. The remaining patients received escalated doses of 64 to 66 Gy, primarily in view of close or positive surgical margins. Reconstruction of the surgical defect was most commonly performed using a pectoralis major myocutaneous (PMMC) flap in 32% of patients, while free flap reconstruction was utilised in 24.3%. Concurrent chemoradiation was administered to 54.4% of the patients, all of whom had either positive surgical margins or ENE. Additionally, eight patients (7.8%) who were considered borderline resectable received neoadjuvant chemotherapy (NACT) to improve resectability and achieve negative margins.

The median interval between surgery and the initiation of RT was 51 days (range: 40–75 days). Adherence to institution‐specific guidelines for CTV and PTV was ensured for all patients. The CTV predominantly included the postoperative tumour bed with a 5–10 mm margin to account for microscopic disease, along with involved nodal regions for high‐risk cases. Cranial nerve pathways were not electively included. This approach focused on minimising treatment‐related toxicity while trying to maintain adequate disease control.

### 3.3 Pattern of Recurrence

At a median follow‐up of 22.2 months (IQR 8–24), 26 patients experienced recurrence. Of the 10 local recurrences, seven experienced in‐field recurrences, predominantly associated with intratumoral PNI, while marginal recurrences were observed in three cases, often linked to extratumoral PNI or large nerve involvement. Advanced pathological stages (pT4 and pN2/N3) and high DoI (DoI ≥ 1.1 cm) were common across all recurrence types, reflecting aggressive disease. Table 3 highlights the recurrence patterns in patients with PNI, focusing on the type of recurrence, PNI characteristics, pathological stage and associated parameters.

**TABLE 3:** Patterns of recurrence with HPR characteristics and recurrence volumes.

Patient	Recurrence type	PNI type	Stage	LVE	DoI (cm)	Margin	Recurrence volume (cc)	Volume within 95% isodose (cc)
1	Marginal recurrence	Large	pT4N0	Present	1.6	Negative	160	117
2	Marginal recurrence	Focal, small, extratumoral	pT4N2	Absent	1.4	Negative	72.7	56.6
3	Infield recurrence	Extensive, intratumoral	pT3N3	Absent	1.5	Negative	42.4	41.8
4	Infield recurrence	Intratumoral	pT3N3	Absent	1.6	Negative	0.4	0.38
5	Infield recurrence	Extratumoral	pT3N3	Absent	2.3	Negative	10.9	10.9
6	Infield recurrence	Extensive, large, intratumoral	pT4N3	Absent	2.1	Negative	45.7	45.6
7	Infield recurrence	Focal, intratumoral	pT4N2	Absent	1.1	Negative	8.1	8
8	Marginal recurrence	Not known	pT4N2	Absent	1.5	Negative	18.4	7.9
9	Infield recurrence	Focal, intratumoral	pT4N3	Absent	1.5	Negative	27.6	26.49
10	Infield recurrence	Focal, intratumoral	pT3N3	Absent	1.1	Close	77.8	77.2

Abbreviations: DoI, depth of invasion; HPR, histopathology report; LVE, lymphovascular emboli; PNI, perineural invasion.

Patterns of failure varied significantly with PNI focality, nerve size and tumour location. In patients with focal PNI, the most common failure type was a combination of local, regional and distant recurrences (49.9%), followed by distant failures (28.6%). In contrast, extensive PNI was more often associated with distant failures (44.4%) and local failures (22.2%), reflecting a more aggressive disease course. These findings suggest that extensive PNI requires targeted strategies to address the higher risk of multifocal and distant failures. For nerve size, large nerve PNI predominantly resulted in distant (40%) and combination failures (40%), while small nerve PNI showed a more even distribution of local, distant and combination failures. Intratumoral PNI was linked to higher combination failure rates (44.4%), whereas extratumoral PNI exclusively led to combination failures, emphasising its aggressive nature.

### 3.4 Example of a Case

A patient with carcinoma of the left lateral border of the tongue underwent a total glossectomy and posterior segmental mandibulectomy with PMMC flap reconstruction. The final histopathology report (HPR) revealed pT4a pN3b disease (Stage IVB). Adjuvant radiation therapy was planned to the postoperative bed and nodal areas with adequate margins as per our standard institutional policy. At a 9‐month follow‐up, the patient presented with local recurrence, confirmed on positron emission tomography‐computed tomography (PET‐CT). Fusion of the recurrence imaging with the initial planning CT demonstrated the recurrence predominantly within the CTV and PTV boundaries, highlighting an in‐field failure. The location of recurrence and dose coverage were analysed, as depicted.

**FIGURE 1:**
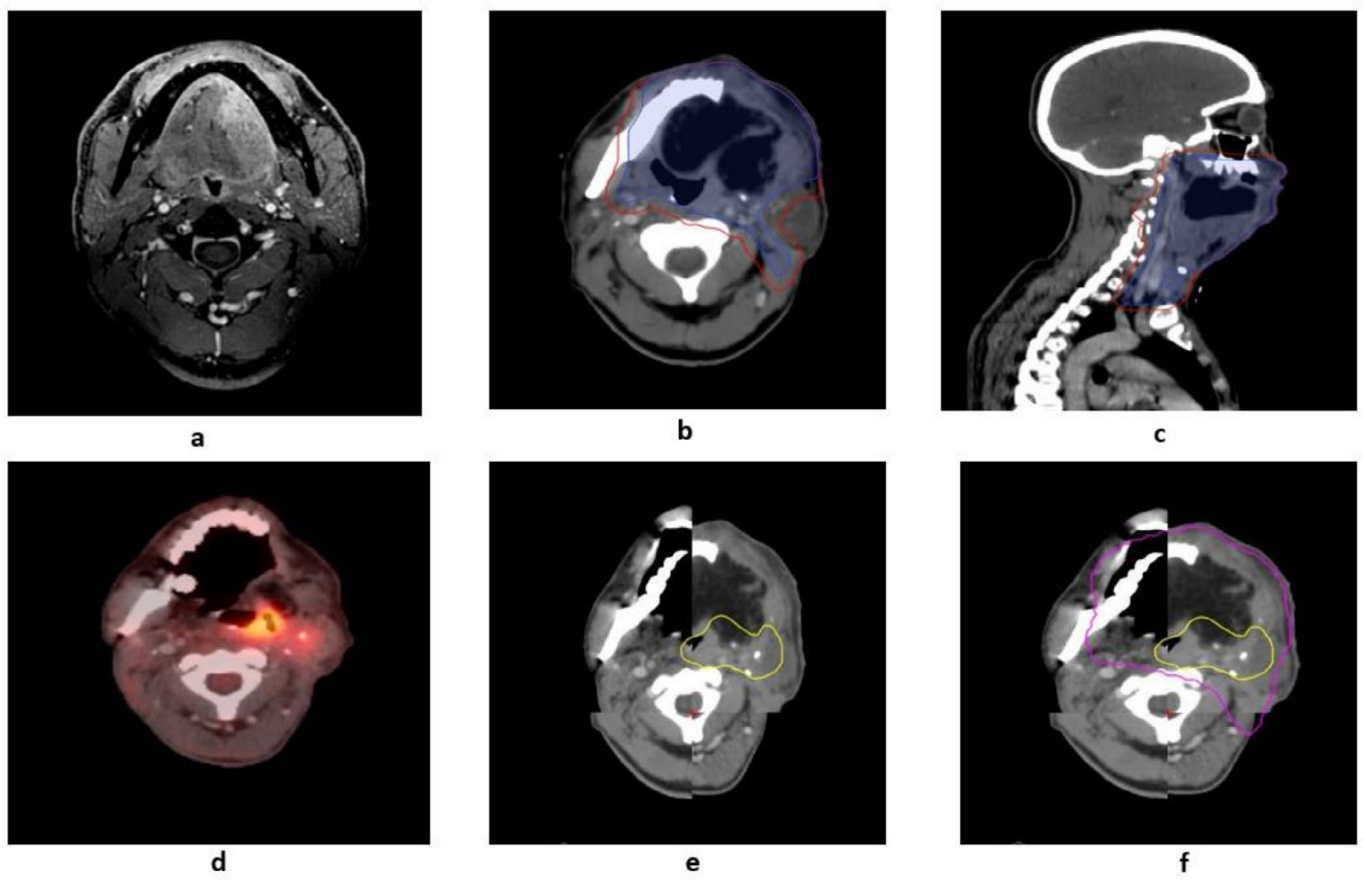
Imaging and recurrence analysis for a case of carcinoma of the left lateral border of the tongue.

Figure 1 illustrates the imaging and recurrence analysis for a case of carcinoma of the left lateral border of the tongue. Panels (a–c) show diagnostic imaging and the delineation of target volumes for adjuvant radiation therapy, with clinical and planning target volumes (CTV and PTV) mapped onto the planning CT. Panel (d) depicts the PET‐CT performed at the time of recurrence, highlighting the metabolic activity of the recurrent tumour. Panel (e) demonstrates the fusion of the recurrence imaging with the initial planning CT, allowing delineation of the recurrence volume on the planning scan. Panel (f) identifies the recurrence pattern as an in‐field failure, with the majority of the recurrence volume falling within the 95% isodose line of the original radiation treatment plan.

### 3.5 Survival Outcomes

**FIGURE 2:**
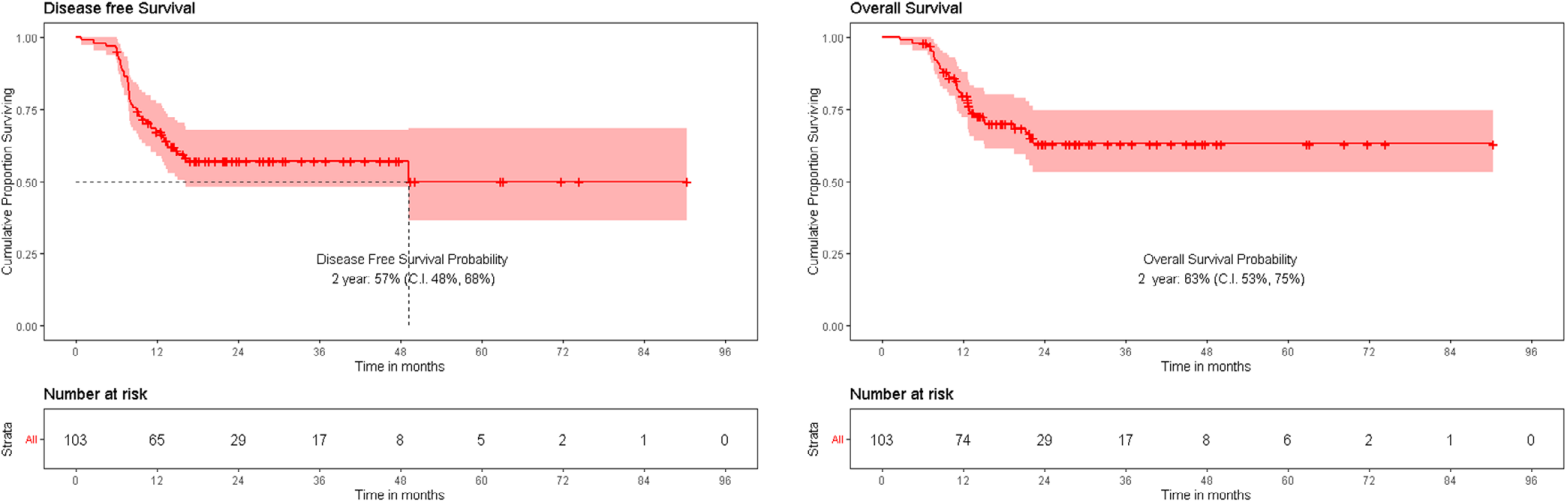
Two‐year OS and DFS of the cohort. DFS, disease‐free survival; OS, overall survival.

At a median follow‐up of 22.21 months, the 2‐year overall survival was 63% (95% CI: 53%–75%) and disease‐free survival was 57% (95% CI: 48%–68%) as shown in Figure 2.

### 3.6 Univariate Analysis for OS

Univariate analysis revealed significant differences in OS based on clinical and pathological factors. The 2‐year OS was significantly lower for advanced tumour stages, with T3/T4 stages (50%) compared to T1/T2 stages (81%) (*p* = 0.0088). Similarly, pathological stage T3/T4 had worse OS (58%) compared to T1/T2 (83%) (*p* = 0.016). Pathological stage grouping also showed worse OS for Stage IV patients (55%) compared to Stage I/II (100%) (*p* = 0.015). Nodal status showed an OS of 38% for N3 compared to 64% for N0, approaching significance (*p* = 0.075), while ENE was associated with significantly lower OS (56% for ENE‐positive vs. 69% for ENE‐negative, *p* = 0.03). DoI > 10 mm had worse OS (56%) compared to DoI ≤ 10 mm (79%) (*p* = 0.027). Other factors, such as histological grade (PDSCC: 50% vs. WDSCC/MDSCC: 70%, *p* = 0.093) and subsite (tongue/floor of mouth: 66% vs. buccoalveolar complex/palate: 61%, *p* = 0.76), showed no statistically significant differences. Age, PNI focality, location and nerve size were also not significantly associated with OS.

### 3.7 Multivariate Analysis for OS

Multivariate analysis identified advanced tumour stage (T3/T4) and ENE as independent predictors of worse overall survival. Patients with T3/T4 stage tumours had a significantly higher risk of death compared to T1/T2 stages, with a hazard ratio (HR) of 2.67 (95% CI: 1.20–5.94, *p* = 0.016). Similarly, the presence of ENE was associated with an increased risk of poor outcomes, with an HR of 2.08 (95% CI: 1.02–4.27, *p* = 0.045). These findings underscore the critical impact of tumour stage and ENE on survival outcomes in this cohort.

### 3.8 Univariate Analysis for DFS

Univariate analysis for DFS demonstrated significant differences based on clinical and pathological factors. Early‐stage disease (Stage I/II) had significantly better DFS at 2 years (74%) compared to advanced stages (44%) (*p* = 0.0023). Pathological T stage also showed better DFS for T1/T2 stages (89%) compared to T3/T4 stages (49%) (*p* = 0.0023). Nodal involvement had a notable impact, with 2‐year DFS ranging from 80% for N0 to 25% for N3, showing statistical significance (*p* = 0.032). Pathological stage grouping indicated the worst DFS for Stage IV (46%) compared to Stage I/II (100%) (*p* = 0.0028). DoI > 10 mm was associated with significantly worse DFS (50%) compared to DoI ≤ 10 mm (84%) (*p* = 0.0053). ENE was linked to worse DFS (50% for ENE‐positive vs. 66% for ENE‐negative), with near statistical significance (*p* = 0.059). Other factors such as histological grade, subsite and age showed no significant differences, while PNI focality, location and nerve size also did not significantly impact DFS.

### 3.9 Multivariate Analysis for DFS

Multivariate analysis identified advanced tumour stage (T3/T4) and DoI as independent predictors of worse DFS. Patients with T3/T4 tumours had a significantly higher risk of recurrence compared to T1/T2 tumours, with a HR of 2.18 (95% CI: 1.07–4.46, *p* = 0.033). DoI ≤ 10 mm was associated with a significantly lower risk of recurrence compared to DoI > 10 mm, with an HR of 0.28 (95% CI: 0.08–0.94, *p* = 0.040). These findings highlight the impact of tumour stage and DoI on DFS in this cohort.

## 4 Discussion

Understanding the patterns of failure in patients who received RT for oral cancers is crucial for optimising RT target volumes, as these significantly influence treatment‐related toxicities and overall patient outcomes. This retrospective study of 103 patients with oral cavity SCC and PNI evaluated the impact of RT volumes on patterns of failure in patients undergoing surgery followed by adjuvant radiotherapy, with or without chemotherapy. Furthermore, the study analysed various patient‐specific, tumour‐specific and treatment‐related factors in the context of PNI to elucidate their collective impact on outcomes.

The median age of the cohort was 48 years, with a substantial proportion (68.9%) under 55 years. This aligns with trends indicating a rising incidence of oral cancers among younger individuals, likely driven by shifts in lifestyle‐related risk factors such as tobacco and alcohol use [18]. A male predominance (79.6%) was noted, consistent with global data linking higher male exposure to these risk factors [19]. Tobacco chewing, reported by 53.4% of the cohort, highlights its etiological role in oral cancers [20]. The most common tumour subsite was the tongue (50.5%), followed by the buccal mucosa (33%), correlating with studies showing a higher incidence of PNI in tongue cancers [21]. The majority of patients presented with advanced disease, with 44.7% having cT4 tumours and 36.9% showing N2 disease. Overall, 64.1% were diagnosed with stage IV disease, reflecting delays in diagnosis and the aggressive nature of tumours in this region.

Pathologically, 45.6% of patients had T4 disease, and 38.8% had N3 disease, indicating extensive nodal metastasis. Pathological Stage IV disease was observed in 71.8% of cases, emphasising the advanced nature of oral cancers treated in this cohort [22]. MDSCC was the predominant histological subtype (64.1%). Reconstruction using the PMMC flap was the most frequent technique (32%). Surgical margins were negative in 88.3%, while close and positive margins were observed in 5.8% each. Most patients (88.3%) received adjuvant RT at a standard dose of 60 Gy in 30 fractions, with around 10% receiving doses up to 64 Gy due to close surgical margins. Delays in starting RT beyond 6 weeks occurred in 23% of patients, largely due to wound complications and machine waiting times, highlighting the need for timely treatment to optimise outcomes.

NACT was administered to 7.8% of patients with borderline resectable disease, aiming to improve resectability and achieve negative margins [23, 24]. While the role of NACT in oral cancers remains debated, its use reflects its potential to improve surgical outcomes [25]. Concurrent chemotherapy was administered in 54.4% of cases, primarily for positive margins (5.8%) or ENE (48.5%), with Cisplatin being the most commonly used agent [26, 27].

Among adverse pathological features, DoI and WPOI stood out as critical prognostic factors. In this cohort, 73.8% had a DoI > 10 mm, and 59.2% were classified as WPOI 4, while 32% were WPOI 5. ENE was present in 48.5% of cases, with 14.6% showing ENE > 2 mm. These findings reaffirm the importance of these high‐risk features in guiding treatment strategies. While Almangush et al. suggested that PNI might not independently affect outcomes when combined with histological risk scores, our study emphasises the distinct prognostic value of PNI [28].

PNI focality emerged as a significant determinant of prognosis. Extensive PNI was associated with higher stages (76.2% were stage IV) and more aggressive tumour behaviour, as noted by its correlation with DoI > 10 mm, WPOI ≥ 4 and locoregional recurrence rates (23.8%). Similarly, nerve size influenced outcomes, with large nerve PNI associated with higher rates of distant and combination failures (40% each). These findings highlight the importance of precise RT targeting in cases involving large nerve PNI to mitigate the risk of metastasis [8]. Intratumoral PNI was linked to combination failures, while extratumoral PNI was more frequently associated with distant recurrences, suggesting distinct biological behaviours based on PNI location [29].

The recurrence pattern analysis showed that 70% of local recurrences were in‐field, reflecting the aggressive nature of the disease rather than inadequacies in RT planning. Marginal recurrences were seen in 30%. Notably, no recurrences were observed at the skull base. While this is a reassuring finding, the absence of a comparator group treated with extended cranial nerve coverage limits definitive conclusions. These findings suggest that, in select patients, a more tailored RT approach may be appropriate; however, further studies are warranted to determine the safety of omitting skull base coverage, particularly in the absence of high‐risk features.

Survival outcomes further confirmed the prognostic impact of advanced tumour stage, ENE and DoI. At a median follow‐up of 22.21 months, the 2‐year OS and DFS were 63% and 57%, respectively. Multivariate analysis revealed that T3/T4 stages and ENE independently predicted worse OS, with hazard ratios (HRs) of 2.67 and 2.08, respectively. For DFS, T3/T4 stages (HR 2.18) and DoI > 10 mm (HR 0.28 for DoI ≤ 10 mm) emerged as significant predictors. These findings highlight the need for aggressive management strategies for high‐risk features while minimising unnecessary toxicity in lower‐risk cases.

Current guidelines, including those from the NCCN, identify PNI as an adverse feature warranting adjuvant RT and recommend the use of conformal techniques when addressing potential perineural spread [30]. However, they do not provide specific directives regarding the extent of cranial nerve coverage. Recent studies suggest that the prognostic relevance of PNI may vary based on quantitative and anatomical features, such as the number of involved foci, nerve diameter and extratumoural versus intratumoural location. These data support the concept that not all PNI carries the same risk and that tailoring RT volumes accordingly could optimise the balance between disease control and toxicity. It cannot be overemphasised that the presence of PNI in the HPR warrants the need for further qualifying the PNI. In this context, the absence of skull base recurrences in our conservatively treated cohort is noteworthy and supports the need to re‐evaluate the routine extension of RT fields to the entire cranial nerve trajectory. Further prospective studies with standardised PNI subclassification and long‐term follow‐up are needed to define optimal RT strategies in this subset [31, 32, 33]. The strengths of our study lie in its comprehensive dataset of 103 patients, enabling detailed analyses and insights into the patterns of failure and outcomes in OSCC with PNI. A major highlight is the effective design of radiotherapy volumes, which resulted in no failures at the skull base, underscoring the adequacy of our RT planning approach. Additionally, the study emphasises the importance of PNI classification by examining various subcategories such as focality, nerve size and location, which provide valuable data for risk stratification and tailored treatment approaches. The high proportion of patients with complete surgical margins (88.3%) ensures a robust foundation for assessing adjuvant therapies.

This study's limitations include its retrospective design, single‐centre setting and the absence of complete PNI subclassification (focality, nerve size and location) in a notable proportion of cases. This limits the robustness and interpretability of subgroup analyses exploring the prognostic impact of specific PNI characteristics. Nonetheless, these findings offer valuable insights into the patterns of failure and prognostic factors in PNI‐positive OSCC, underscoring the importance of personalised treatment strategies. Future research should focus on prospective, multicentre studies with long‐term follow‐up and quality‐of‐life assessments to validate these findings and refine RT guidelines.

## 5 Conclusion

Our study highlights the complexities of managing oral cavity squamous cell carcinomas with PNI. While extensive PNI is clearly linked to more aggressive disease behaviour and poorer outcomes, our findings suggest that expanding radiotherapy volumes to cover the entire course of involved nerves may not necessarily improve patient outcomes. Instead, such an approach may increase the risk of treatment‐related toxicities. These results emphasise the importance of a personalised treatment strategy that carefully considers the extent of PNI, the size and location of nerve involvement and other high‐risk pathological features. Furthermore, our study underscores the critical need for detailed reporting of PNI characteristics in pathology reports, particularly when PNI is the sole adverse feature influencing treatment decisions.

Future research should focus on refining risk stratification models to better identify patients who may benefit from more intensive adjuvant treatments, such as higher radiation doses, concurrent chemotherapy or novel therapeutic approaches. Additionally, there is a need to further explore strategies that balance treatment intensity with patient safety, aiming to optimise survival outcomes while minimising treatment‐related morbidities. The ultimate goal should be to improve not only the survival rates but also the quality of life for this unique and high‐risk patient population.

## Author Contributions

**Sarbani Ghosh Laskar:** conceptualization (lead), data curation (equal), formal analysis (equal), funding acquisition (equal), investigation (equal), methodology (equal), project administration (lead), resources (equal), software (equal), supervision (equal), validation (equal), visualization (equal), writing – original draft (equal), writing – review and editing (equal). **Anuj Kumar:** conceptualization (equal), data curation (equal), formal analysis (equal), investigation (equal), methodology (equal), resources (equal), software (equal), supervision (equal), writing – original draft (equal), writing – review and editing (equal). **Ashwini Adhau:** formal analysis (equal), methodology (equal), resources (equal), software (equal), writing – original draft (equal). **Shwetabh Sinha:** data curation (equal), investigation (equal), resources (equal), writing – review and editing (equal). **Samarpita Mohanty:** data curation (equal), investigation (equal), resources (equal), writing – review and editing (equal). **Munita Bal:** data curation (equal), investigation (equal), resources (equal), writing – review and editing (equal). **Neha Mittal:** data curation (equal), investigation (equal), resources (equal), writing – review and editing (equal). **Swapnil Rane:** data curation (equal), investigation (equal), resources (equal), writing – review and editing (equal). **Asawari Patil:** data curation (equal), investigation (equal), resources (equal), writing – review and editing (equal). **Ashwini Budrukkar:** data curation (equal), investigation (equal), resources (equal), writing – review and editing (equal). **Monali Swain:** data curation (equal), investigation (equal), resources (equal), writing – review and editing (equal). **Pallavi Rane:** data curation (equal), methodology (equal), resources (equal), writing – review and editing (equal). **Gouri Pantvaidya:** data curation (equal), investigation (equal), resources (equal), writing – review and editing (equal). **Sudhir Nair:** data curation (equal), investigation (equal), resources (equal), writing – review and editing (equal). **Deepa Nair:** data curation (equal), investigation (equal), resources (equal), writing – review and editing (equal). **Anuja Deshmukh:** data curation (equal), investigation (equal), resources (equal), writing – review and editing (equal). **Shivakumar Thiagarajan:** data curation (equal), investigation (equal), resources (equal), writing – review and editing (equal). **Richa Vaish:** data curation (equal), investigation (equal), resources (equal), writing – review and editing (equal). **Vidisha Tuljapurkar:** data curation (equal), investigation (equal), resources (equal), writing – review and editing (equal). **Chandrashekhar Dravid:** data curation (equal), investigation (equal), resources (equal), writing – review and editing (equal). **Poonam Joshi:** data curation (equal), investigation (equal), resources (equal), writing – review and editing (equal). **Rathan Shetty:** data curation (equal), investigation (equal), resources (equal), writing – review and editing (equal). **Arjun Singh:** data curation (equal), investigation (equal), resources (equal), writing – review and editing (equal). **Pankaj Chaturvedi:** data curation (equal), investigation (equal), resources (equal), writing – review and editing (equal).

## Conflicts of Interest

The authors declare no conflicts of interest.

## Data Availability

The data supporting this study will not be publicly shared, as no data‐sharing plan was included at the time of institutional ethics approval. However, data may be made available upon reasonable request to the corresponding author.

## References

[1] P. Chaturvedi , S. Byakodi , S. Hiremath , et al., “Oral Cancer in India: Epidemiological and Clinical Aspects,” Journal of the Indian Medical Association 113, no. 7 (2015): 37–42.

[2] Ministry of Health and Family Welfare , “Global Adult Tobacco Survey, India, 2017,” 2017.

[3] GLOBOCAN , “GLOBOCAN 2022: Global Cancer Statistics,” 2022, https://gco.iarc.fr/.

[4] M. Evans , P. Bonomo , P. C. Chan , et al., “Post‐Operative Radiotherapy for Oral Cavity Squamous Cell Carcinoma: Review of the Data Guiding the Selection and the Delineation of Post‐Operative Target Volumes,” Radiotherapy and Oncology 207 (2025): 110880. 40194704 10.1016/j.radonc.2025.110880

[5] J. G. Batsakis , “Nerves and Neurotropic Carcinomas,” Annals of Otology, Rhinology, and Laryngology 94, no. 4 Pt 1 (1985): 426–427. 4026129

[6] M. Brandwein‐Gensler , M. Teixeira , M. S. Lewis , et al., “Predicting Outcome in Oral Squamous Cell Carcinoma,” American Journal of Surgical Pathology 29, no. 2 (2005): 167–178. 15644773 10.1097/01.pas.0000149687.90710.21

[7] K. A. Kurtz , H. T. Hoffman , M. B. Zimmerman , and R. A. Robinson , “Perineural and Vascular Invasion in Oral Cavity Squamous Carcinoma: Increased Incidence on Re‐Review of Slides and by Using Immunohistochemical Enhancement,” Archives of Pathology & Laboratory Medicine 129, no. 3 (2005): 354–359. 15737030 10.5858/2005-129-354-PAVIIO

[8] C. Liebig , G. Ayala , J. A. Wilks , D. H. Berger , and D. Albo , “Perineural Invasion in Cancer: A Review of the Literature,” Cancer 115, no. 15 (2009): 3379–3391. 19484787 10.1002/cncr.24396

[9] F. M. Paes , A. D. Singer , A. N. Checkver , R. A. Palmquist , G. de la Vega , and C. Sidani , “Perineural Spread in Head and Neck Malignancies: Clinical Significance and Evaluation With 18F‐FDG PET/CT,” Radiographics 33, no. 6 (2013): 1717–1736. 24108559 10.1148/rg.336135501

[10] S. B. Chinn , M. E. Spector , E. L. Bellile , et al., “Impact of Perineural Invasion in the Pathologically N0 Neck in Oral Cavity Squamous Cell Carcinoma,” Otolaryngology—Head and Neck Surgery (United States) 149, no. 6 (2013): 893–899. 10.1177/0194599813506867 PMC4118458 24154744

[11] M. E. Miller , B. Palla , Q. Chen , et al., “A Novel Classification System for Perineural Invasion in Noncutaneous Head and Neck Squamous Cell Carcinoma: Histologic Subcategories and Patient Outcomes,” American Journal of Otolaryngology 33, no. 2 (2012): 212–215, 10.1016/j.amjoto.2011.06.003. 22177613

[12] H. C. Ko , V. Gupta , W. F. Mourad , et al., “A Contouring Guide for Head and Neck Cancers With Perineural Invasion,” Practical Radiation Oncology 4, no. 6 (2014): e247–e258. 25407876 10.1016/j.prro.2014.02.001

[13] P. Gorayski , M. Foote , S. Porceddu , and M. Poulsen , “The Role of Postoperative Radiotherapy for Large Nerve Perineural Spread of Cancer of the Head and Neck,” Journal of Neurological Surgery. Part B, Skull Base 77, no. 2 (2016): 173–181. 27123394 10.1055/s-0036-1571839 PMC4846398

[14] R. L. Bakst , C. M. Glastonbury , U. Parvathaneni , et al., “Perineural Invasion and Perineural Tumor Spread in Head and Neck Cancer,” International Journal of Radiation Oncology, Biology, Physics 103, no. 5 (2019): 1109–1124. 30562546 10.1016/j.ijrobp.2018.12.009

[15] A. J. Holcomb , N. Farrokhian , C. Tolan , et al., “Adjuvant Radiotherapy Mitigates Impact of Perineural Invasion on Oncologic Outcomes in Early‐Stage Oral Cavity Squamous Cell Carcinoma: A Multi‐Institutional Analysis of 557 Patients,” Oral Oncology 142 (2023): 106420. 37182430 10.1016/j.oraloncology.2023.106420 PMC10575471

[16] A. K. Anand , P. Agarwal , A. Gulia , et al., “Significance of Perineural Invasion in Locally Advanced Bucco Alveolar Complex Carcinomas Treated With Surgery and Postoperative Radiation ± Concurrent Chemotherapy,” Head & Neck 39, no. 7 (2017): 1446–1453. 28452191 10.1002/hed.24792

[17] L. A. Dawson , Y. Anzai , L. Marsh , et al., “Patterns of Local‐Regional Recurrence Following Parotid‐Sparing Conformal and Segmental Intensity‐Modulated Radiotherapy for Head and Neck Cancer,” International Journal of Radiation Oncology, Biology, Physics 46, no. 5 (2000): 1117–1126, 10.1016/s0360-3016(99)00550-7. 10725621

[18] S. Sharma , L. Satyanarayana , S. Asthana , K. K. Shivalingesh , B. S. Goutham , and S. Ramachandra , “Oral Cancer Statistics in India on the Basis of First Report of 29 Population‐Based Cancer Registries,” Journal of Oral and Maxillofacial Pathology 22, no. 1 (2018): 18–26. 29731552 10.4103/jomfp.JOMFP_113_17 PMC5917535

[19] Y.‐C. Lee , C.‐K. Young , H.‐T. Chien , et al., “Characteristics and Outcome Differences in Male and Female Oral Cavity Cancer Patients in Taiwan,” Medicine (Baltimore) 100, no. 44 (2021): e27674. 34871246 10.1097/MD.0000000000027674 PMC8568378

[20] C. Rivera , “Essentials of Oral Cancer,” International Journal of Clinical and Experimental Pathology 8, no. 9 (2015): 11884–11894. 26617944 PMC4637760

[21] D. Nair , M. Mair , H. Singhvi , et al., “Perineural Invasion: Independent Prognostic Factor in Oral Cancer That Warrants Adjuvant Treatment,” Head & Neck 40, no. 8 (2018): 1780–1787. 29707840 10.1002/hed.25170

[22] N. Mummudi , J. P. Agarwal , S. Chatterjee , I. Mallick , and S. Ghosh‐Laskar , “Oral Cavity Cancer in the Indian Subcontinent—Challenges and Opportunities,” Clinical Oncology 31, no. 8 (2019): 520–528, 10.1016/j.clon.2019.05.013. 31174947

[23] V. Noronha , A. Dhanawat , V. M. Patil , et al., “Long‐Term Outcomes of Neo‐Adjuvant Chemotherapy on Borderline Resectable Oral Cavity Cancers: Real‐World Data of 3266 Patients and Implications for Clinical Practice,” Oral Oncology 148 (2024): 106633, 10.1016/j.oraloncology.2023.106633. 37988838

[24] V. M. Patil , K. Prabhash , V. Noronha , et al., “Neoadjuvant Chemotherapy Followed by Surgery in Very Locally Advanced Technically Unresectable Oral Cavity Cancers,” Oral Oncology 50, no. 10 (2014): 1000–1004, 10.1016/j.oraloncology.2014.07.015. 25130412

[25] J. Bernier , C. Domenge , M. Ozsahin , et al., “Postoperative Irradiation With or Without Concomitant Chemotherapy for Locally Advanced Head and Neck Cancer,” New England Journal of Medicine 350, no. 19 (2004): 1945–1952, 10.1056/NEJMoa032641. 15128894

[26] J. S. Cooper , Q. Zhang , T. F. Pajak , et al., “Long‐Term Follow‐Up of the RTOG 9501/Intergroup Phase III Trial: Postoperative Concurrent Radiation Therapy and Chemotherapy in High‐Risk Squamous Cell Carcinoma of the Head and Neck,” International Journal of Radiation Oncology, Biology, Physics 84, no. 5 (2012): 1198–1205, 10.1016/j.ijrobp.2012.05.008. 22749632 PMC3465463

[27] A. C. Chi , N. Katabi , H.‐S. Chen , and Y.‐S. L. Cheng , “Interobserver Variation Among Pathologists in Evaluating Perineural Invasion for Oral Squamous Cell Carcinoma,” Head and Neck Pathology 10, no. 4 (2016): 451–464. 27140176 10.1007/s12105-016-0722-9 PMC5082046

[28] A. lmangush , I. O. Bello , H. Keski‐Säntti , et al., “Depth of Invasion, Tumor Budding, and Worst Pattern of Invasion: Prognostic Indicators in Early‐Stage Oral Tongue Cancer,” Head and Neck 36, no. 6 (2014): 811–818. 23696499 10.1002/hed.23380 PMC4229066

[29] L.‐Y. Lee , D. De Paz , C.‐Y. Lin , et al., “Prognostic Impact of Extratumoral Perineural Invasion in Patients With Oral Cavity Squamous Cell Carcinoma,” Cancer Medicine 8, no. 14 (2019): 6185–6194. 31290283 10.1002/cam4.2392 PMC6797567

[30] National Comprehensive Cancer Network , “NCCN Clinical Practice Guidelines in Oncology (NCCN Guidelines): Head and Neck Cancers,” Version 2.2025, https://www.nccn.org/professionals/physician_gls/pdf/head‐and‐neck.pdf.

[31] H. W. Cheng , L. H. Lin , H. P. Lin , and C. J. Liu , “Perineural Invasion Unveiled: Deciphering the Prognostic Impact of Diameter and Quantity Subcategories in Oral Cancer,” Journal of Otolaryngology ‐ Head & Neck Surgery 54 (2025): 19160216251316219, 10.1177/19160216251316219. 39902557 PMC11792026

[32] S. Mohamed , D. Callanan , P. Sheahan , and L. Feeley , “Significance of Location and Extent of Perineural Invasion in Early‐Stage Oral Cavity Squamous Cell Carcinoma,” Histopathology 86, no. 6 (2025): 993–1000, 10.1111/his.15406. 39762203 PMC11964579

[33] S. Purohit , P. Ahlawat , S. Tandon , A. Jain , and M. Gairola , “Challenges Seen With Perineural Invasion in Head and Neck Cancer—A Review,” Oral Oncology Reports 6 (2023): 100028, 10.1016/j.oor.2023.100028.

